# Diagnostic criteria for left ventricular non-compaction in cardiac computed tomography

**DOI:** 10.1371/journal.pone.0235751

**Published:** 2020-07-10

**Authors:** Tobias A. Fuchs, Ladina Erhart, Jelena R. Ghadri, Bernhard A. Herzog, Andreas Giannopoulos, Ronny R. Buechel, Simon F. Stämpfli, Christiane Gruner, Aju P. Pazhenkottil, Markus Niemann, Philipp A. Kaufmann, Felix C. Tanner

**Affiliations:** 1 Department of Cardiology, University Heart Center, Zurich, Switzerland; 2 Department of Nuclear Medicine, Cardiac Imaging, University Hospital Zurich, Zurich, Switzerland; 3 Heart Clinic Luzern, Luzern, Switzerland; 4 Faculty Mechanical and Medical Engineering, Furtwangen University, Villingen-Schwenningen, Germany; Univeristy of Tennessee, UNITED STATES

## Abstract

**Purpose:**

Left ventricular non-compaction (LVNC) is characterized by a 2-layered myocardium composed of a noncompacted (NC) and a compacted (C) layer. The echocardiographic NC:C ratio is difficult to assess in many patients. The aim of the study was to assess the value of cardiac computed tomography (CCT) for the diagnosis of LVNC.

**Methods:**

In this prospective controlled study, segmental analysis of transthoracic echocardiography (TTE) and prospective ECG-triggered CCT was performed in 17 patients with LVNC and 19 healthy controls. In TTE maximal NC and C thickness was measured at enddiastole and endsystole in the segment with most prominent trabeculation in short axis views. In CCT, maximal segmental NC and C thickness was measured during diastole, and NC:C ratio was determined. Spearman’s correlation coefficient and receiver operating characteristic curves were calculated.

**Results:**

The median [IQR] radiation dose was 1.3[1.2–1.5]mSv. The CCT thickness of the C layer was significantly lower in patients with LVNC as compared to controls in the inferolateral, midventricular, lateral-, inferior-, and septal-apical segments. The CCT NC:C ratio differed significantly between LVNC and controls in the inferior-midventricular and all the apical segments. NC:C ratio correlated significantly between TTE and CCT at enddiastole (*σ* = 0.8) and endsystole (*σ* = 0.9). Using a CCT NC:C ratio ≥1.8, all LVNC patients could be identified.

**Conclusion:**

LVNC can be diagnosed with ECG-triggered low-dose CCT and discriminated from normal individuals using a NC:C ratio of ≥1.8 in diastole. There is a very good correlation of NC:C ratio in TTE and CCT.

## Introduction

Isolated left ventricular noncompaction (LVNC) is characterized by a two-layered myocardium consisting of a noncompacted (NC) and a compacted layer (C). Echocardiographic criteria with a maximal endsystolic ratio of NC:C > 2 and deep intertrabecular recesses with a predominant location in the apical segments and absence of coexisting cardiac abnormalities have been established and validated against anatomical examination of the heart [1]. However, these criteria are sometimes difficult to assess due to insufficient echocardiographic windows. Complementary imaging modalities such as cardiac magnetic resonance (CMR) or cardiac computed tomography (CCT) might overcome such limitations and provide an added diagnostic value.

CT offers a high spatial resolution and is independent of any acoustic window. Moreover, the acquisition of three-dimensional datasets allows interpretation and measurements of any angle post scanning. Additionally, technical refinements, such as prospective ECG-triggering and iterative reconstruction algorithms have led to substantial radiation dose reduction [2, 3] and have made CCT feasible with a radiation exposure < 1mSv. So far, only limited data with small groups of patients are available regarding the diagnosis of LVNC in CCT and to our knowledge, no study has investigated the feasibility to diagnose LVNC using low-dose prospectively triggered CCT. Melendez-Ramirez et al. included 10 LVNC patients in their study and patients were exposed to a high effective radiation exposure of 12 mSv [4]. The group of Sidhu et al. investigated 8 patients with LVNC and used retrospective gating similar to the previous study [5]. In order to use CT as a complementary modality for diagnosis of LVNC, morphological CCT criteria are crucial. Thus, it was the aim of the present prospective controlled study to establish CCT criteria that distinguish LVNC from healthy controls by prospective ECG-triggered low-dose CCT using TTE as the standard of reference.

## Materials and methods

### Patient population

**Fig 1:**
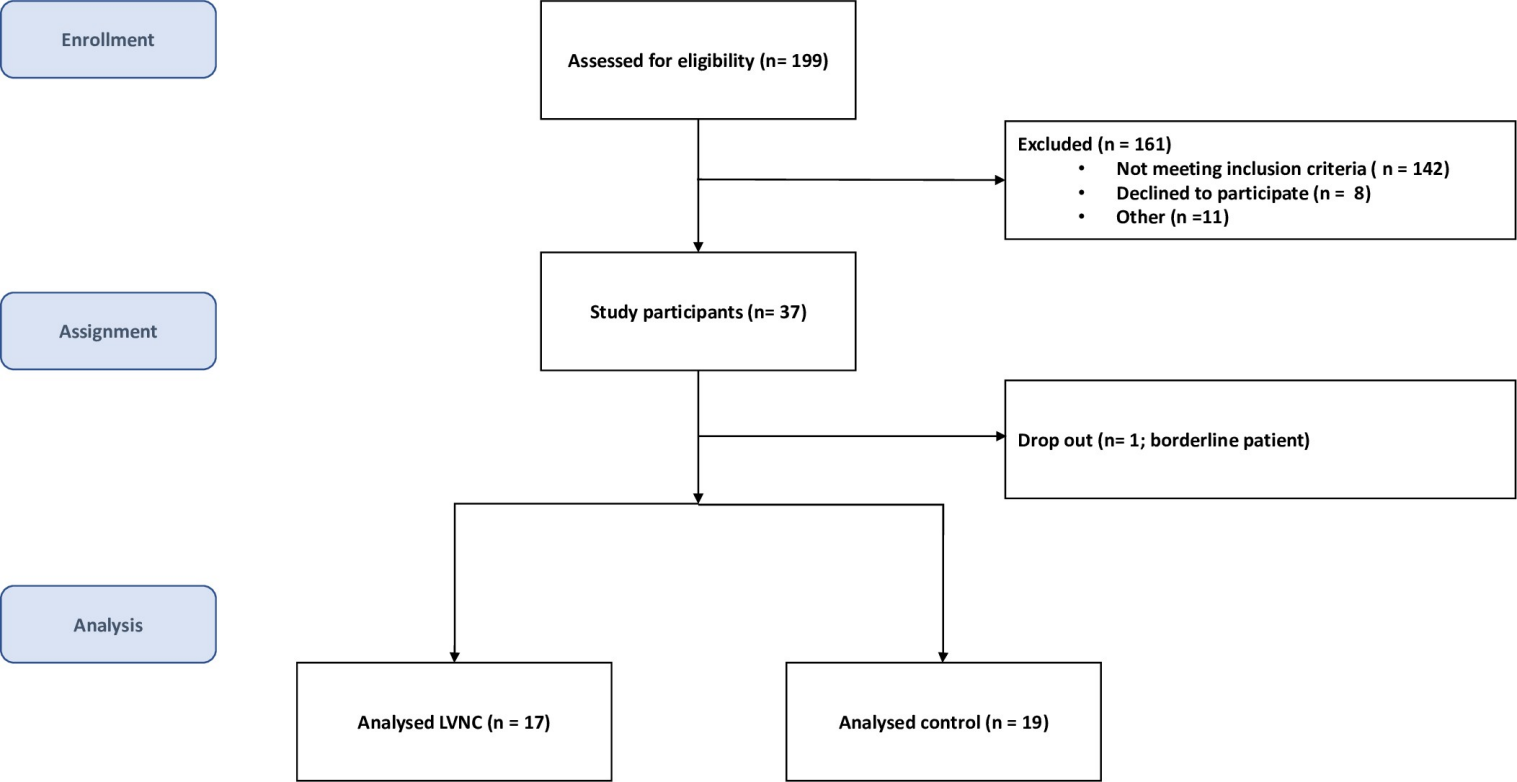
Participant flow chart.

In this prospective controlled study, thirty-six patients were included to undergo TTE and prospectively ECG-triggered low dose CCT at the University Hospital in Zurich. After screening our database, 37 participants were recruited via phone call between October 2011 and December 2014. The final study population consisted of 19 healthy controls (volunteers who were recruited via phone call) and 17 patients with definite diagnosis of LVNC (Fig 1). All these individuals were Caucasians. Median [IQR] age was 54 [43–58] years, 24 (67%) were males, and the median [IQR] BMI was 25 [23–27] kg/m^2^. The diagnosis of LVNC was established by TTE with strict adherence to previously published criteria comprising the following four conditions: (1) absence of coexisting cardiac abnormalities; (2) a two-layered myocardial wall with a C subepicardial layer and a NC subendocardial layer, with a maximal end-systolic ratio of NC:C > 2 in parasternal short-axis view; (3) a predominant mid-lateral, mid-inferior, and apical location of affected segments; and (4) colour Doppler with adjustment of the colour scale for evidence of deep intertrabecular recesses filled with blood from the left ventricular cavity. Additionally, compacted wall thickness ≤ 8.1 mm was found to be very specific for myocardial thickening in LVNC compared to normal controls [1, 6].

Patients with renal insufficiency (GFR <60ml/min; 2.5%), known allergy to iodinated contrast agent, known hyperthyroidism, non-sinus rhythm, high burden of premature ventricular or supraventricular beats (> 10/min), or hypotension were excluded. Furthermore, patients with any cardiac disease other than isolated LVNC were excluded. This study complies with the declaration of Helsinki, the study protocol was approved by the local ethics committee (Kantonale Ethikkommission Zürich), and was registered at ClinicalTrials.gov (NCT01470014). Written informed consent was obtained from all patients.

### Transthoracic echocardiography

TTE was performed using commercially available equipment (Siemens Medical Solutions USA, Inc., Malvern, PA; Philips iE33, Philips Medical Systems, Andover, MA; and GE Vivid 7, GE Healthcare, Milwaukee, WI). Images acquired in parasternal long-axis, parasternal short-axis, and apical views were analysed offline on digitally stored loops.

In all participants, TTE analysis was performed by an experienced observer who determined wall thickness from digitally stored loops for this study blinded to the CCT. Each segment was analysed in standard parasternal short axis views (2D) regarding the presence of LVNC. Maximal thickness of NC layer in LVNC patients or trabeculated layer in controls, respectively, and maximal thickness of C layer were measured in the segment with most prominent recesses (in LVNC) or trabeculations (in controls) at end-systole because the two layers are then easier to distinguish and because the ratio of the two layers was established by a side-by-side comparison of echocardiographic images with the corresponding pathologic specimens as previously described [1]. Additionally, maximal thickness of NC and C layers was measured in end-diastole in these segments. At the midventricular level, segments with papillary muscles were not used for measurements to avoid any confounding effects. The ratio of maximal thickness of NC and C was calculated. To facilitate reading, both the NC layer in patients with LVNC and the trabeculated layer in controls will be called ‘‘noncompacta” throughout the text, while both the compacted layer in patients with LVNC and the nontrabeculated layer in controls will be called ‘‘compacta” throughout the text. Each echo and measurement were performed by two observers in consensus. One of these observers was an experienced imaging specialist for all the examinations.

### Cardiac computed tomography

**Fig 2:**
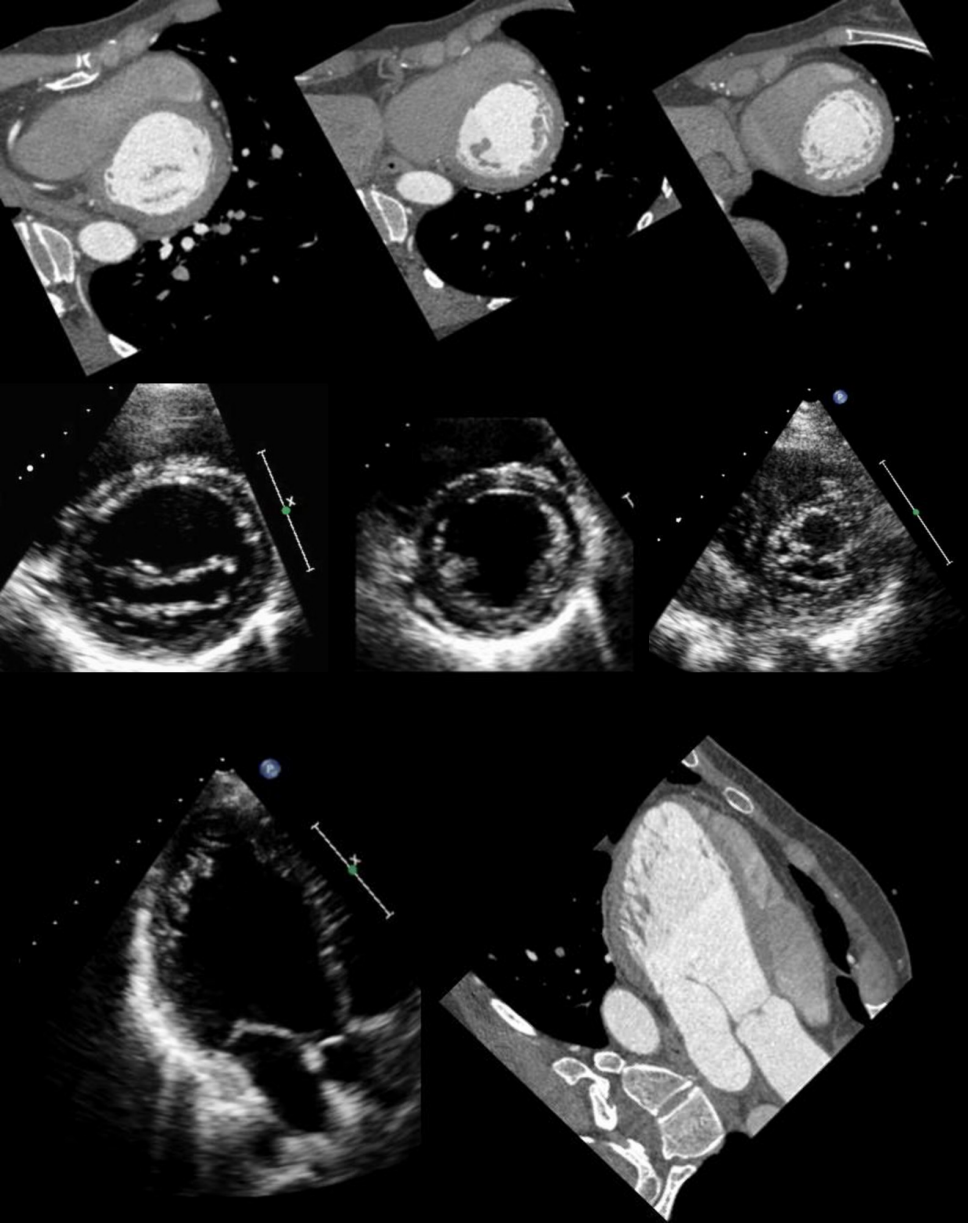
TTE and contrast enhanced CCT. Basal, midventricular and apical short axis and three-chamber view of a patient with isolated LVNC acquired by transthoracic echocardiography and prospectively ECG-triggered low dose CCT.

Prior to the CCT examination metoprolol (AstraZeneca, London, UK) up to 20mg was injected intravenously if heart rate was higher than 65 beats per minute in order to obtain optimal image quality for CCT. All patients underwent contrast-enhanced CCT during inspiration breath hold with prospective ECG-triggering as previously reported on a 64-slice CT scanner (Discovery CT 750 High Definition; GE Healthcare, Milwaukee, WI, USA; Fig 2). By choosing the smallest possible window at only one distinct end-diastolic phase of the RR-cycle (i.e. 75%), we ascertained the lowest achievable effective dose delivery, using a commercially available protocol (SnapShot Pulse, GE Healthcare). [3, 7]. Iodixanol (Visipaque 320, 320 mg/ml, GE Healthcare) was injected into an antecubital vein followed by 50 ml saline solution. Contrast media volume (40–105 ml) and flow rate (3.5–5 ml/s) were adapted to body surface area (BSA) as previously validated [8]. Scanning parameters were as follows: Collimation of 64 x 0.625 mm, gantry rotation time of 0.35 s and field of view was 25 cm. Tube voltage (100–120 kV) and tube current (450–700 mA) were adapted to body mass index as previously described [7, 9]. Effective radiation dose from CCTA was calculated as the product of dose-length product (DLP) times a conversion coefficient for chest (k = 0.014 mSv mGy-1 cm-1) [10].

All images were transferred to an external workstation (AW 4.6, GE Healthcare) for analysis. Measurements were performed by an experienced CCT reader, blinded to echocardiographic results. Cardiac planes were obtained at short axis views (basal, midventricular and apical), and long axis views (two-, three, and four-chamber view). The 17-segment model was used as proposed by the American Heart Association, and the European Association of Cardiovascular Imaging [11, 12]. Maximal thickness of C and NC layer was measured in each segment in short axis views (basal, midventricular and apical) and the ratio of NC: C was calculated. At the midventricular level, papillary muscles were carefully tracked in order to avoid any confounding effects. The apex (segment 17) was excluded from the analysis because this segment would exhibit an artificially high NC: C ratio due to a thin C and a thick NC layer, as previously suggested [4].

### Statistical analysis

Quantitative variables were expressed as mean±standard deviation or median and interquartile range [IQR]. The statistical software package SPSS 20.0 (SPSS, Chicago, IL) was used for analysis. Segmental CCT NC:C ratio was calculated and compared between LVNC and control. Intra and inter-rater agreement for the CCT based assessment of NC:C ratio were measured using Bland Altmann analysis for each segment in a representative sample of 12 patients (6 NC and 6 controls). The mean difference (bias) and Bland-Altman (BA) limits of agreement (i.e., mean ± 1.96 SD) were calculated. The data were tested for normal distribution by Shapiro-Wilk test. Comparison of variables with no normal distribution was performed with Mann-Whitney U Test. Spearman’s correlation coefficient was calculated between TTE and CCT segmental ratios and BA limits of agreement were calculated. Receiver operating characteristic curves were used to generate cut-off values for distinguishing LVNC from control on a per-segment and a per-patient basis. The optimal cut-point were determined by the Youden-Index. Patients with at least one segment fulfilling the established cut-off value were considered as LVNC patients. P values of less than 0.05 were considered statistically significant. A sample size of 411 segments was calculated in order to provide a desired precision of 6%.

## Results

A total of 17 patients with LVNC and 19 controls underwent low-dose CCT without any adverse events and were included in the final analysis. Previous to CCT the patients received 3 [0–10] mg metoprolol and the median [IQR] radiation exposure of low-dose CCT was 1.3 [1.2–1.5] mSv. The median [IQR] heart rate during the scan was 56 [50–61] beats per minute. Patient baseline characteristics of both groups are listed in Table 1.

**Table 1:** Patient baseline characteristics.

	LVNC	Control	
Number (n)	17	19	p = 0.74
Age (years)	53 [41–62]	54 [49–58]	p = 0.96
Male (%)	71	63	p = 0.046
Smoking (%)	24	26	p = 0.003
Hypertension (%)	23	5	p < 0.001
Dyslipidaemia (%)	18	16	p < 0.001
Diabetes (%)	6	11	p < 0.001
Family History of LVNC or SCD	47	0	p < 0.001
CRT-D or ICD	29	0	p < 0.001
Oral anticoagulation	47	0	p < 0.001
BSA (m^2^)	1.8 [1.6–2.0]	1.9 [1.8–2.0]	p = 0.12
BMI (kg/m^2^)	24 [21–25]	25 [24–27]	p = 0.32
Contrast media (ml)	70 [60–80]	60 [60–80]	p = 0.64
CCT radiation dose (mSv)	1.3 [1.2–1.5]	1.4 [1.2–1.6]	p = 0.48
LVEF (%)	52 [44–57]	59 [57–63]	p = 0.002
LVESV (ml)	51 [39–72]	43 [34–47]	p = 0.05
LVEDV (ml)	104 [88–143]	105 [92–116]	p = 0.70
LVEDD (mm)	51 [48–58]	48 [47–52]	p = 0.02
LVESD (mm)	35 [33–39]	31 [27–32]	p < 0.001
RV Area D (cm2)	17 [15–21]	19 [15–20]	p = 0.76
TAPSE (mm)	22 [19–25]	22 [18–23]	p = 0.33
FAC (%)	43 [38–47]	42 [39–43]	p = 0.56

Data are given in median [IQR] were appropriate. LVEF = left ventricular ejection fraction; LVESV = left ventricular end-systolic volume; LVEDV = left ventricular end-diastolic volume; LVEDD = left ventricle end-diastolic diameter; LVESD = left ventricle end-systolic diameter; RV = right ventricle; TAPSE = Tricuspid annular plane systolic excursion; FAC = fractional area change. D = Diastole. LVNC = left ventricular non-compaction, SCD = sudden cardiac death; CRT = Cardiac resynchronization therapy; ICD = implantable cardiac defibrillator.

Based on echocardiographic myocardial layer thickness (Table 2), the maximal myocardial median [IQR] NC:C ratio for LVNC vs controls was 3.3 [2.5–4.3] vs 0.9 [0.6–1.1] (p<0.001) during end-systole and 4.3 [3.1–5.1] vs 1.0 [0.8–1.5] (p<0.001) during end-diastole.

**Table 2:** Echocardiographic myocardial layer thickness.

Echocardiographic median [IQR] myocardial layer thickness (mm)	LVNC	Controls	p value
NC (end-systolic)	15.0 [12–16.5]	7 [6–9]	p < 0.001
C (end-systolic)	5.0 [3.5–5]	9 [7–10]	p < 0.001
NC:C ratio (end-systolic)	3.3 [2.5–4.3]	0.9 [0.6–1.1]	p < 0.001
NC (end-diastolic)	14.0 [12.5–16]	6 [5–9]	p < 0.001
C (end-diastolic)	3.0 [3.0–4.5]	6 [5–7]	p < 0.001
NC:C (end-diastolic)	4.3 [3.1–5.1]	1 [0.8–1.5]	p < 0.001

Echocardiographic median [IQR] myocardial layer thickness (mm) of the segment with maximal values per patient for end-systole and end-diastole. NC: non-compacta, C: compacta.

The CCT median value for maximal diastolic NC thickness per patient was significantly higher in LVNC vs controls. This resulted in a significantly higher median NC:C ratio in LVNC vs controls (Table 3). Intra- and inter-observer agreement for CCT based evaluation of the NC:C ratio, was excellent with small bias and narrow BA limits of agreement (bias 0.016 and 0.001 with limits of agreement -0.21, +0.24 and -0.13 to +0.14, for inter-rater and intra-rater agreement respectively).

**Table 3:** CCT myocardial layer thickness.

CCT median [IQR] myocardial layer thickness (mm)	LVNC	Controls	p value
NC	12.9 [11.4–15.3]	4.5 [0–9.3]	p < 0.001
C	6.3 [5.8–6.7]	7.8 [6.9–8.1]	p = 0.001
NC:C ratio	2.2 [1.8–2.4]	0.7 [0–1.1]	p < 0.001

CCT median [IQR] myocardial layer thickness (mm) of the segment with maximal values per patient. NC: non-compacta, C: compacta.

**Fig 3:**
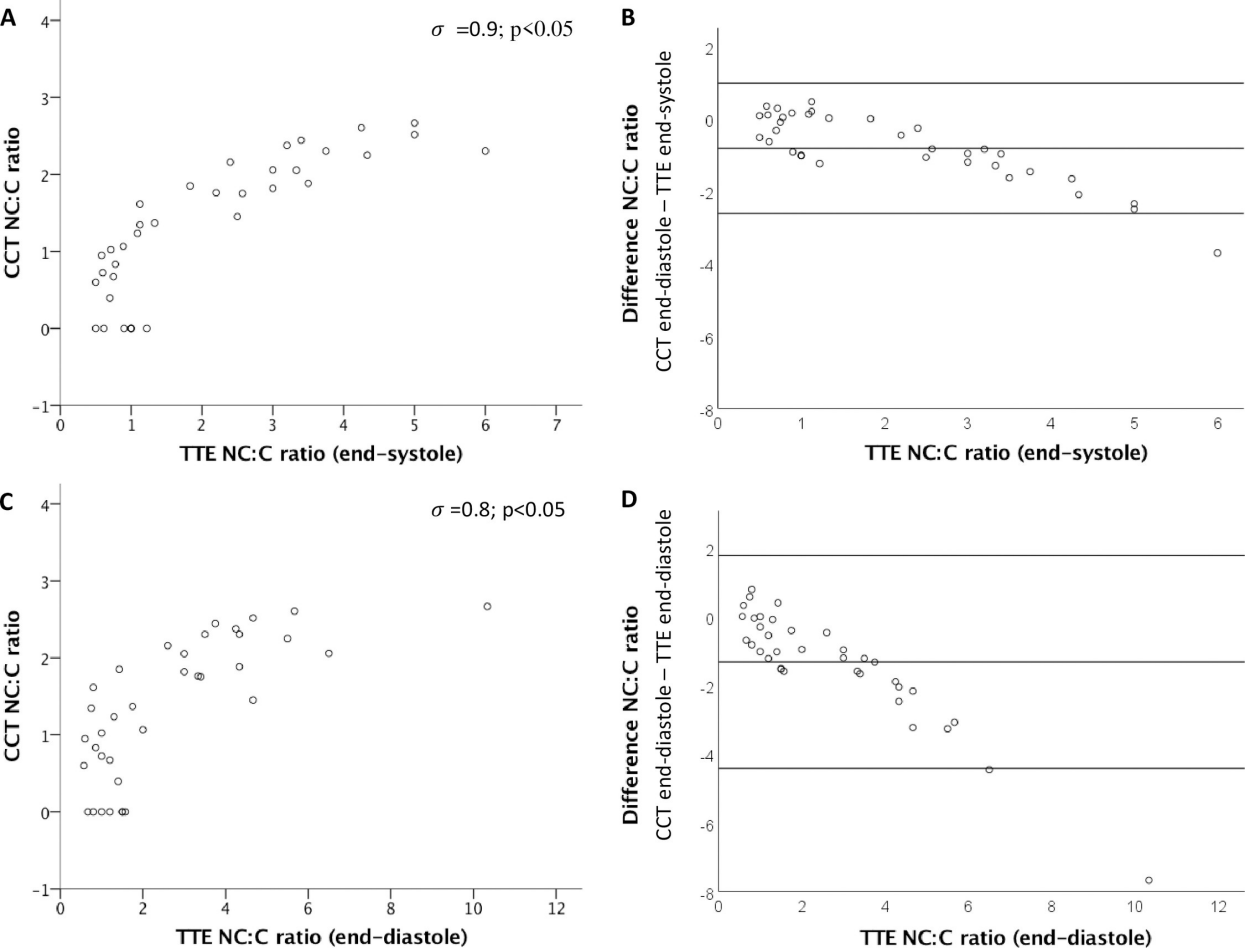
Comparison of the NC:C ratio between CCT and TTE. Comparison between NC:C ratio between CCT and TTE reveal a significant correlation both at end-systole (*σ* = 0.9; A) and end-diastole (*σ* = 0.8; C). Also Bland Altman limits of agreement are presented regarding the comparison of NC:C ratio for CCT and TTE at end-systole (-2.6 to 1; B) and end-diastole (-4.4 to 1.8; D).

There was a significant correlation of the NC:C ratio between CCT and TTE both at endsystole (*σ* = 0.9; p<0.001) with narrow Bland-Altman limits of agreement (-2.6 to 1.0) and at enddiastole (*σ* = 0.8; p<0.001) with broader Bland-Altman limits of agreement (-4.4 to 1.8) (Fig 3).

**Fig 4:**
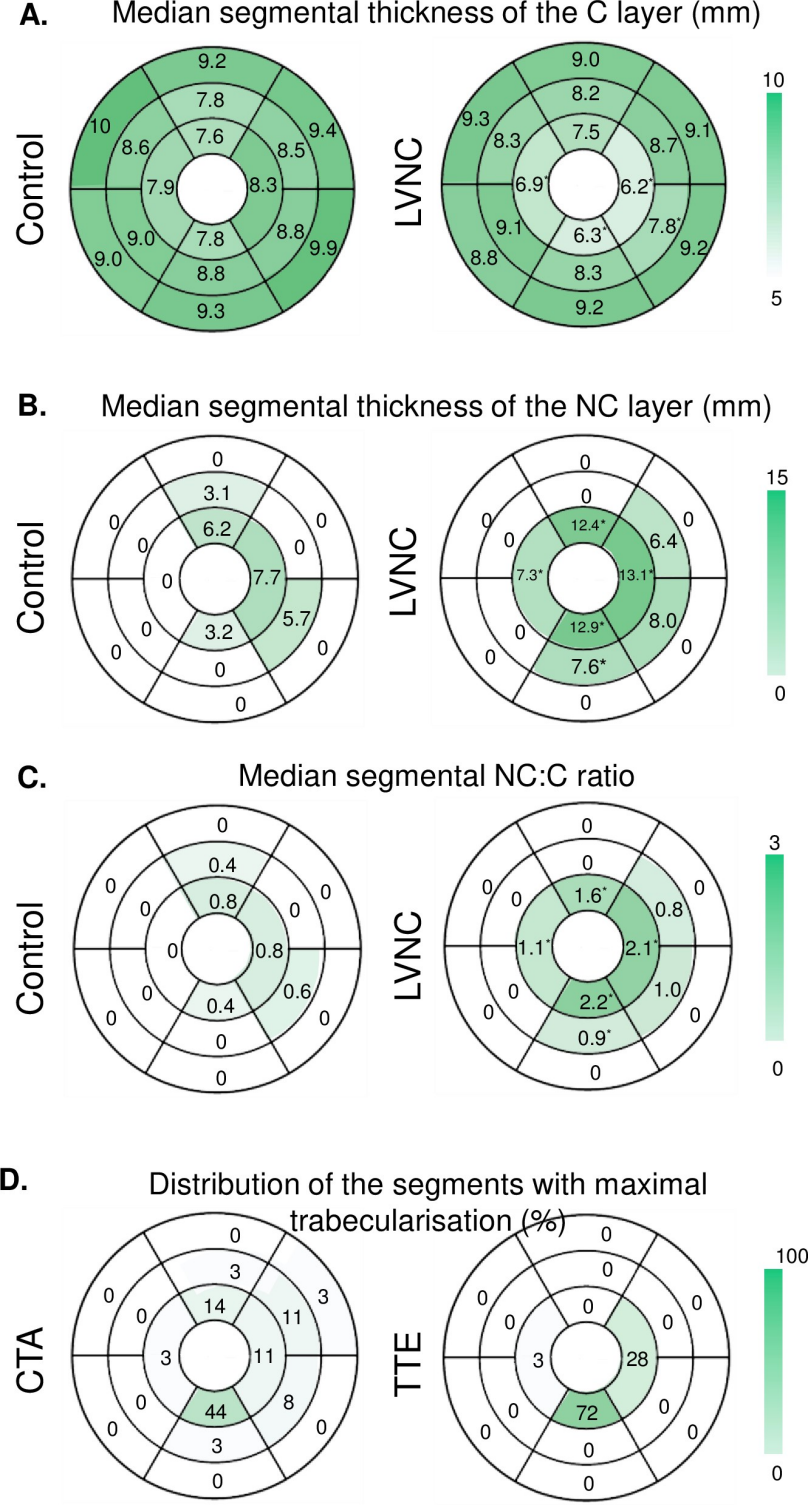
Polar plots. The polar plots are illustrating the median CCT segmental thickness of the C layer (mm) for the control (left) and LVNC (right) group (A). Basal segments 1–6 are shown in the outer layer, midventricular segments 7–12 in the middle layer and apical segment 13–16 in the inner layer (counterclockwise). The CCT segmental median thickness of the C layer was significantly lower in the LVNC group compared to controls in the inferolateral-midventricular, lateral-, inferior-, and septal-apical segments (*), whereas it did not differ significantly in the remaining segments. The polar plots are illustrating the median CCT segmental thickness of the NC layer (mm) for the control (left) and LVNC (right) group (B). Basal segments 1–6 are shown in the outer layer, midventricular segments 7–12 in the middle layer and apical segment 13–16 in the inner layer (counterclockwise). The CCT segmental median thickness of the NC layer was significantly higher in the LVNC group compared to controls in the inferoior-midventricular, anterior-apical, septal-apical, and inferior-apical, and latero-apical, segments (*), whereas it did not differ significantly in the remaining segments. The polar plots are illustrating the median CCT segmental NC:C ratio for the control (left) and LVNC (right) group (C). Basal segments one to six are shown in the outer layer, midventricular segments 7–12 in the middle layer and apical segment 13–16 in the inner layer (counterclockwise). The median CCT NC:C ratio differed significantly between LVNC and controls in the inferior-midventricular, lateral-, inferior-, septal-, and anterior-apical segments(*). The polar plots are illustrating the segments with maximal trabecularisation (%) for CCT (left) and TTE (right) (D). Basal segments one to six are shown in the outer layer, midventricular segments 7–12 in the middle layer and apical segment 13–16 in the inner layer (counterclockwise). The inferior-apical segment exhibited the most prominent trabeculations in both TTE (72%) and CCT (44%).

The CCT segmental median [IQR] thickness of the C layer was significantly lower in LVNC compared to controls in the inferolateral-midventricular (7.8 [6.8–9.8] mm vs 8.8 [7.8–9.5] mm), lateral-apical (6.2 [5.9–8.0] mm vs 8.3 [7.5–9.3] mm), inferior-apical (6.3 [5.7–6.7] mm vs 7.8 [6.9–8.4] mm), and septal-apical (6.9 [6.0–7.5] mm vs 7.9 [7.2–8.8] mm) segments, whereas it did not differ significantly in the remaining segments (Fig 4A).

The CCT segmental median [IQR] thickness of the NC layer was significantly higher in LVNC compared to controls in the inferior-midventricular (7.6 [0–12.9] mm vs 0 [0–0] mm), anterior-apical (12.4 [3.1–14.2] mm vs 6.2 [0–8.2] mm), septal-apical (7.3 [0–13.1] mm vs 0 [0–0] mm), and inferior-apical (12.9 [11.1–15.3] mm vs 3.2 [0–8.2] mm), and lateral-apical (13.1 [11.3–15.0] mm vs 7.7 [4.9–8.4] mm), segments, whereas it did not differ significantly in the remaining segments (Fig 4B).

The CCT median NC:C ratio differed significantly between LVNC and controls in the inferior-midventricular (0.9 [0–2.1] vs 0 [0–0]), lateral-apical (2.1 [1.8–2.3] vs 0.8 [0.6–1.1]), inferior-apical (2.2 [1.8–2.5] vs 0.4 [0.0–1.0]), septal-apical (1.1 [0–2.1] vs 0 [0–0]), and anterior-apical (1.6 [0.3–2.1] vs 0.8 [0–1.0]) segments (Fig 4C). Accordingly, the inferior-apical segment exhibited the most prominent trabeculations in both TTE and CCT as it represented maximal trabeculation in 72% and 44% of the patients, respectively (Fig 4D).

**Fig 5:**
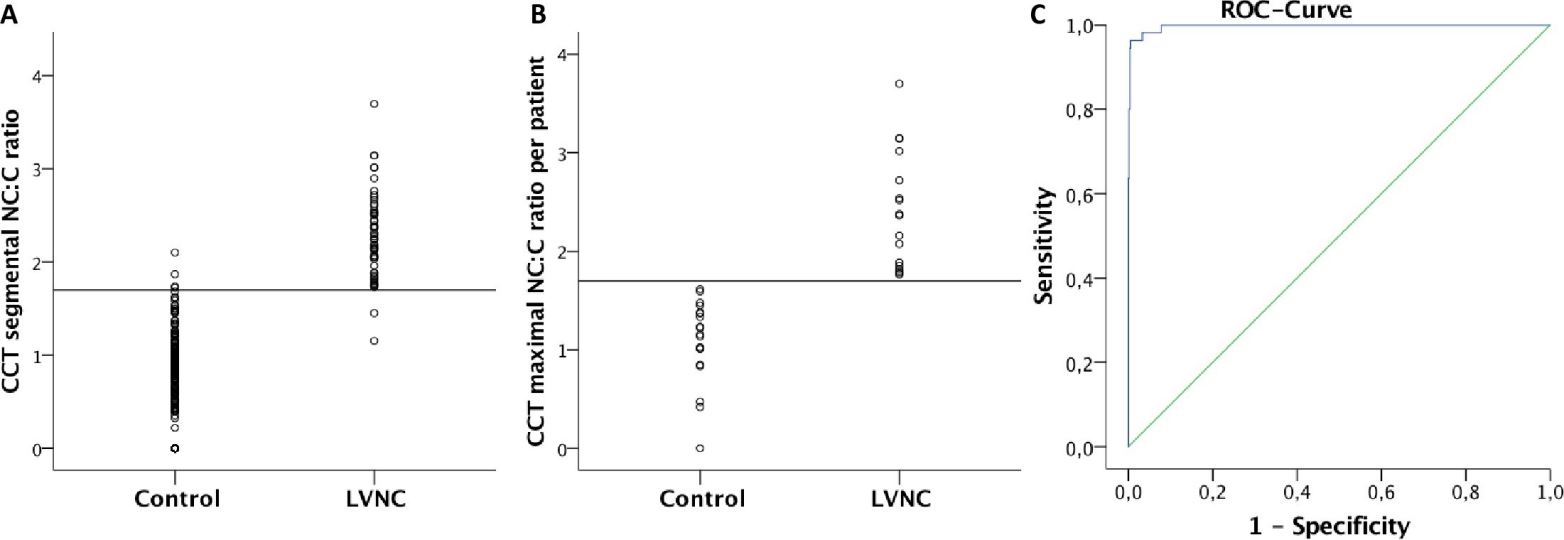
CCT NC:C ratio per patient and per segment. Using the NC:C threshold of ≥1.8 (horizontal line), CCT identified 55 LVNC segments in 17 patients. 3 segments were false positive, and 2 segments were false negative (A). Using this cut-off, all LVNC patients could be identified (B). ROC analysis of the quantitative segmental NC:C CCT analysis revealed an area under the curve of 0.997 (C).

Based on echocardiography, 54 affected segments were identified in the 17 LVNC patients and 0 affected segments in the 19 controls, respectively. The quantitative segmental NC:C CCT analysis revealed a optimal cut-off value of ≥1.8 for detecting an affected LVNC segment with an area under the curve of 0.997 (Fig 5).

## Discussion

This study demonstrates the feasibility to diagnose LVNC with prospective ECG triggered low-dose CCT in a prospective controlled trial with a median [IQR] radiation dose of 1.3 [1.2–1.5] mSv.

Since its first description by Engberding et al in 1984, LVNC has gained increasing attention [13]. Based on morphological criteria established for echocardiography [1, 14–16], it is categorized as an unclassified cardiomyopathy by the European Society of Cardiology [17]. Accurate diagnosis of LVNC is essential as patients may suffer from complications such as heart failure, ventricular arrhythmias, sudden cardiac death, and systemic embolic events [18]. Until now most accepted criteria are based on echocardiography; however, good image quality is mandatory for applying these criteria because accurate measurements in parasternal short axis views should only be performed with correct angulation. Oblique views will necessarily lead to overestimation of myocardial wall thickness and possibly misinterpretation of images resulting in a false diagnosis of LVNC. In this study, CCT short axis and long axis views were used in order to (a) assess maximal thickness of the C and the NC layer and (b) carefully avoid inccidental measurement of papillary muscles as part of the NC layer. While Sidhu et al used long axis views only [5], the present study took full advantage of the strength of CCT which consists of a three-dimensional dataset with high spatial resolution and allows post-processing along any angulation including short axis views analogous to those generated by echocardiography.

The low prevalence of LVNC indicated between 0.02% to 0.26% in adults [19–21] may have contributed to the fact that so far only case reports [22–24] and two original articles with 8 and 10 LVNC patients, respectively, have investigated the accuracy of CCT in patients with this cardiomyopathy [4, 5]. In the present study, we have prospectively enrolled a cohort of 36 individuals including 17 patients with previously diagnosed LVNC. The cut-off value of ≥1.8 to distinguish normal from pathological myocardial segments was lower as compared to the previous studies reporting a cut-off value of ≥ 2.2 for CCT [4, 5]. This might be related to the observation that LVNC was analysed at a later stage of the disease in the previous studies resulting in more dilated left ventricles [4, 5]. Howevere, left ventricular ejection fraction continuisly decreased in a substantial number of our participants and implantaple cardioverter defibrillator or cardiac resynchronization therapy was necessary. Additionally, while both Melendez et al [4] and Sidhu et al [5] acquired CCT with retrospective gating, we used prospective ECG triggered CCT acquired in the 75% of the RR interval. Different phases might result in different C and NC measurement and different cut-off values. Furthermore, comparability between the CCT derived NC:C thresholds from the former studies and our ratio might be difficult due to the low number of patients included in the studies. Phenotypic variability might indeed result in different CCT derived NC:C thresholds and a higher patient number could overcome this issue. Our cohort included 36 patients including 17 patients with LVNC, representing the largest CCT LVNC cohort so far. However, further studies are needed to expand the patient number and confirm these observations.

A maximal endsystolic ratio of NC:C >2 has been established as one of the major criteria to diagnose LVNC by TTE and validated versus anatomical examination of the heart [1]. Compared to echocardiography, our CCT NC:C threshold of ≥1.8 NC:C is somewhat lower. However, different modalities provide different values due to different spatial resolution and the differences in the techniques itself. Furthermore, in contrast to our measurements, which were performed in diastole in short and long axis, Jenni et al measured the echocardiographic ratio of NC:C in parasternal short axis views during end-systole because the two layers are easier to distinguish by echocardiography in this phase of the cardiac cycle and because the ratio of the two layers was established by a side-by-side comparison of echocardiographic images with the corresponding pathologic specimens [1]. In contrast, Chin et al used the ratio of myocardial thickness from the epicardial surface to through and epicardial surface to peak of the trabeculation at end-diastole in parasternal long axis and four chamber view [14]. The phase of the measurements in the studies by Stöllberger and Belanger is less well characterized [15, 16]. In this study, we measured the NC:C ratio in echocardiography both at end-systole and end-diastole and observed an excellent correlation of NC:C ratio obtained by CTA with both echocardiographic measurements. The correlation between diastolic NC:C ratio in CTA and end-systolic NC:C ratio in echocardiography was higher than end-diastolic ratio in the latter modality, which is possibly related to the better echocardiographic differentiation of the two layers in end-systole.

Several different methods have been proposed to diagnose LVNC by CMR. Peterson et al applied an end-diastolic NC:C Ratio ≥2.3 measured in long axis cine views at the site with most pronounced trabeculations [25]. Stacey et al defined an end-systolic ratio ≥2 in short axis views to diagnose LVNC [26]. Captur et al described an end-diastolic loss of the base to apex fractal dimension gradient ≥1.3 for LVNC [27] and Jacquier used short axis views to measure trabecular mass [28]. The fact that no single method has been asserted oneself supports the need of further modalities as well as additional criteria such as clinical and/or genetic information. On the other hand additional information on fibrosis burden can be provided by CMR which is crucial for arrhythmia risk stratification. Our CCT threshold differed from CMR values. Differences in spatial resolution of the two modalities, measurements in long axis versus short axis, and variability in the time frame of measurements might have contributed to the different cut-off values.

Any diagnostic tool should offer a positive balance of harms and benefits. This is particularly true for CCT, which has in the past evoked a vivid discussion on its potential carcinogenic risk and its justification for a purely diagnostic test. Melendez-Ramirez et al and Sidhu et al reported a high accuracy to detect LVNC in CCT; however, their CCT resulted in a high radiation exposure for the patients of >12 mSv, whereas this value averaged 1.3 [1.2–1.5]mSv despite high accuracy in our study. This was enabled by prospective ECG triggering, e.g. limiting the beam to a narrow diastolic phase, and a BMI adapted scanning protocol, with adjective tube current and voltage [3]. In fact, both previously published studies used only the diastolic phase for diagnosis of LVNC despite scanning throughout the whole heart cycle. We have demonstrated for the first time, that diagnosis of LVNC is feasible with prospectively ECG-triggered CCT resulting in a low radiation dose.

Based on our results CCT can be used complementary to previously established imaging techniques. However, overdiagnosis of LVNC is an issue of concern. Zemrak et al demonstrated in the MESA (Multi-Ethnic Study of Atherosclerosis) study that a huge number of asymptomatic patients fulfilled the Peterson CMR criteria for LVNC without exhibiting any adverse events [29]. This observation illustrates that additional criteria are essentially needed in order to confirm the diagnosis of LVNC. Gebhard et al demonstrated that a maximal systolic C thickness <8 mm allows to differentiate LVNC from normal hearts and from hypertrophic ventricles as well using TTE [6]. The present results are in line with this observation as the mean diastolic thickness of the C layer in CCT was significantly lower in patients with LVNC in the inferolateral-midventricular as well as the lateral-, inferior- and septal-apical segments. Furthermore, the echocardiographic C layer thickened in end-systole as compared to end-diastole in controls, whereas it did not in LVNC patients. On top of additional imaging criteria, information such as genotype [30], evidence of LVNC related clinical events, and a positive family history for LVNC or other cardiomyopathies should be included when LVNC is diagnosed in an individual [31].

Recently, iodine staining enhanced micro CT enabled the differentiation between the NC and C layer in mice with deletion of the cell cycle inhibitor protein p27^Kip1^ [32] and tissue characterization might be feasible in the near future by the use of dual-source or single-source dual energy techniques [7]. Hence, systematic clinical application of CCT for diagnosis of LVNC might become common practice in the future.

There are some potential limitations of this study. Despite the prospectively controlled study design, the cohort size was small and the results were obtained in a single center. Multi-center studies are thus needed to confirm the results in a larger and unselected population. In this study, we have established the NC:C threshold only in LVNC patients versus healthy controls. Thus, the applicability of the proposed criterion will have to be validated in the differentiation of LVNC from other cardiac diseases such as patients with hypertrophic or dilated cardiomyopathy. Furthermore, African-Americans and athletes were not included in the study as it is known that they are more likely to fulfill the LVNC criteria. Finally, clinical history regarding arrhythmia was not available.

To conclude, this study demonstrates that CCT allows to discriminate LVNC patients from healthy persons with low radiation exposure. CCT might be used complementary to echocardiography in patients with equivocal results or poor image quality to provide further evidence whether the patient is affected or not, particularly when there is an indication to exclude the presence of coronary artery disease in such an individual. Furthermore, as patients undergo CCT for other reasons, this threshold may be used for answering the question whether accidentally seen trabeculations are consistent with the diagnosis of LVNC.

## Supporting information

S1 Checklist TREND checklist. (PDF)

S1 File Ethical protocol. (DOC)
