# Supplementary material for: Diagnostic criteria for left ventricular non-compaction in cardiac computed tomography
Source: PLoS One. 2020 Jul 10;15(7):e0235751. doi: 10.1371/journal.pone.0235751 (PMC7351212; doi:10.1371/journal.pone.0235751)
Supplement: S1 File — (DOC) [file pone.0235751.s002.doc]

**Cardiac Computed Tomography: Characteristics of Isolated Left Ventricular Non-compaction**

Cardiology and Cardiac Imaging, University Hospital of Zurich

Sponsor - Investigator:

Prof. Dr. med. Felix Tanner

Rämistrasse 100

8091 Zürich

Tel.: 044 255 3999

Fax: 044 255 4859

E-mail: felix.tanner@usz.ch

| **STUDY PROTOCOL** | |  |
| --- | --- | --- |
|  |  | |
| Study No.: | 2011-0099 | |
| Title: | Cardiac Computed Tomography: Characteristics of Isolated Left Ventricular Non-compaction | |
| Clinical Phase: | Study with intervention | |
| Sponsor | University Hospital Zürich | |
| Primary Investigator: | Prof. Dr. med. F. Tanner | |
| Institute /  Departments: | Division of Cardiology, CardioVascular Center, University Hospital Zürich, 8091 Zürich | |
| Date of Protocol: | 08.06.2011 | |
| Version | 1.3 | |
| Planned Dates of Study: | August 2011 – July 2013 | |
| GCP Statement | **The study will be conducted in accordance with the ethical principles that have their origin in the Declaration of Helsinki and are consistent with ICH Good Clinical Practice as well as regulatory requirements.** | |
| Confidential | **This protocol is the property of the Department of Cardiology (University Hospital Zurich), and may not - in full or in part - be passed on, reproduced, published or otherwise used without permission.** | |

SYNOPSIS

| **Study Title** | Cardiac Computed Tomography: Characteristics of Isolated Left Ventricular Non-compaction |
| --- | --- |
| **Planned study period:** | August 2011 – July 2013 |
| **Study Objectives:** | We aim to test the diagnostic accuracy of cardiac CT in distinguishing pathological IVNC from lesser degrees of trabecular layering seen in those with cardiomyopathies and concentric left ventricular hypertrophy, potential differential diagnoses. |
| **Study Design:** | Single centre intervention study |
| **Number of subjects:** | 40 |
| **Number of centre’s:** | 1 |
| **Assessment of:** | Ratio of non-compacted to compacted myocardium |

**TABLE OF CONTENTS**

1 LIst of abbreviations [5](#__RefHeading___Toc125181301)

2 Investigational site staff [6](#__RefHeading___Toc125181302)

2.1 Principle Investigator: [6](#__RefHeading___Toc125181303)

2.2 Co-investigators: [6](#__RefHeading___Toc125181304)

2.3 Study Coordinators: [7](#__RefHeading___Toc125181305)

3 BackGROUND INFORMATION 9

4 OBJECTIVES AND PURPOSE 10

5 design OF THE TRIAL [11](#__RefHeading___Toc125181312)

6 SeLECTION OF TRIAL SUBJECTS 12

6.1 Recruitment 12

6.2 Inclusion criteria 12

6.3 Exclusion criteria 12

7 Methods 13

7.1. Cardiac Computed Tomography and Data analysis 13

7.2 Echocardiography and Data analysis 13

7.2 Cardiovascular Magnetic Resonance 14

8 ASSESSMENT OF SAfETY 15

9 STATISTICS 16

10 Duties ON THE PART OF THE INVESTIGATOR 17

11 ethical considerations 18

12 Quality and quality assurance 19

13 Insurance 20

14 SIGNATURE PAGE 21

15 REFERENCES 22

# LIst of abbreviations

| CT | Computed tomography |
| --- | --- |
| IVNC | Isolated left ventricular non-compaction |
| CMR | Cardiovascular Magnetic Resonance |
| WHO | World health organization |

# Investigational site staff

## Principle Investigator:

Prof. Dr. med. F. Tanner,

Division of Cardiology, University Hospital Zürich

Rämistrasse 100, CH-8091 Zurich

Phone: 044 255 3999

Fax: 044 255 4859

E-mail: felix.tanner@usz.ch

**2.2 Co-investigators:**

| Prof. Dr. med. Philipp Kaufmann  Division of Radiology/ Cardiac Imaging  University Hospital Zurich  Rämistrasse 100  CH-8091 Zurich  Tel: 044 255 4196  Fax: 044 255 4859  E-mail: [pak@usz.ch](mailto:frank.enseleit@usz.ch) | Dr. med. Bernhard Herzog  Division of Cardiology  University Hospital Zurich  Rämistrasse 100  CH-8091 Zurich  Tel: 044 255 2928  Fax: 044 255 4414  E-mail: [bernhard.herzog@usz.ch](mailto:bernhard.herzog@usz.ch) |
| --- | --- |
| Fr. Dr. med. Jelena Ghadri  Division of Radiology/ Cardiac Imaging  University Hospital Zurich  Rämistrasse 100  CH-8091 Zurich  Tel: 044 255 2928  Fax: 044 255 4414  E-mail: [Jelena-Rima.Ghadri@usz.ch](mailto:Jelena-Rima.Ghadri@usz.ch) | Dr. med. Michael Fiechter  Division of Radiology/ Cardiac Imaging  University Hospital Zurich  Rämistrasse 100  CH-8091 Zurich  Tel: 044 255 2928  Fax: 044 255 4414  E-mail: [michael.fiechter@usz.ch](mailto:michael.fiechter@usz.ch) |

Dr. med. Tobias Fuchs

Division of Radiology/ Cardiac Imaging

University Hospital Zurich

Rämistrasse 100

CH-8091 Zurich

Tel: 044 255 2928

Fax: 044 255 4414

E-mail: [Tobias. Fuchs@usz.ch](mailto:Tobias. Fuchs@usz.ch)

## Study Coordinators:

Dr. med. Bernhard Herzog / Fr. Dr. med. Jelena Ghadri

Division of Cardiology / Division Radiology Cardiac Imaging

University Hospital Zurich

Rämistrasse 100

CH-8091 Zurich

Tel: 044 255 2928

Fax: 044 255 4414

E-mail: [bernhard.herzog@usz.ch](mailto:bernhard.herzog@usz.ch)

E-mail: [jelena-rima.ghadri@usz.ch](mailto:jelena-rima.ghadri@usz.ch)

# BACKGROUND INFORMATION

Cardiomyopathy is a heart muscle disease that can affect people of all ages and is in many cases inherited. People suffering from this disorder are often at risk of arrhythmia or sudden cardiac death or both. Until now, cardiomyopathies are not curable and diagnosis is often challenging. WHO and the international Society and Federation of Cardiology Task Force on the Definition and Classification of Cardiomyopathies distinguish five major disease entities.

The category of unclassified cardiomyopathies is certainly unsatisfying, as it reflects the lack of understanding of diseases that are not yet fully accessible to current diagnostic tools. Isolated ventricular non-compaction (IVNC) is, according to the WHO`s classification an unclassified cardiomyopathy (1) that was firstly described 1984 by its typical appearance of a thin compacted outer layer (epicardial) and a much thicker non-compacted inner (endocardial) layer in echocardiography (2).

Recently, an epidemiological study of primary cardiomyophathies in Australian children has shown that 9.2% of all cases identified IVNC as the third most frequent cardiomyopathy after dilated and hypertrophic cardiomyopathy (3).

Echocardiography or in the recent years cardiovascular magnetic resonance (CMR) were considered as reference of standard for the diagnosis of IVNC and facilitates the diagnosis. However, in echocardiography cases of poor image quality often exist; therefore, contrast echocardiography can help demarcating the endocardial borders which may facilitate to establish the diagnosis (4) and in CMR metallic implants, time consuming protocols and claustrophobia are limiting factors. Cardiac computed tomography (CT) has rapidly evolved into an accurate noninvasive assessment of cardiac disorders. Using a new scanning protocol (prospective ECG triggering) results in massive reduction in radiation dose from 20 – 25 mSv to 0.8 – 3.0 mSv. CT findings for IVNC have been rarely described in the literature. There is only one study by Hamamichi et al who investigated six adolescent patients by using ultrafast CT and magnetic resonance imaging to diagnose IVNC (5). CT showed early defects of varying degrees and late enhancement of the non-compacted myocardium. Diagnostic criteria for IVNC have not been established in cardiac CT.

# OBJECTIVES AND PURPOSE

Although the diagnostic criteria for IVNC have been well established for echocardiography and CMR, such validation is lacking for CT. Such criteria are of great clinical relevance as a growing number of patients undergo a cardiac CT scan in daily clinical routine.

We aim to test the diagnostic accuracy of cardiac CT in distinguishing IVNC from lesser degrees of trabecular layering seen in potential differential diagnoses such as cardiomyopathies and left ventricular hypertrophy or dilation.

We hypothesize that echochardiographic diagnosed pathological trabeculation can be distinguished by determining the ratio of non-compacted to compacted myocardium on cardiac CT.

# design OF THE TRIAL

This is a prospective single-center study with intervention in 40 subjects. All patients will undergo a cardiac CT, an echocardiography and CMR within 3 month. This trial does not include any follow-up visit.

Endpoint: Identification of IVNC with the ratio of non-compacted to compacted myocardium.

# Selection of trial Subjects

## Recruitment

Patients will be recruited from the Department of Cardiology and Cardiac Imaging Division. They will be asked if they want to participate in this study.

## Inclusion criteria

- Established diagnosis of IVNC or differential diagnosis as listed above.
- Age: Men ≥18 years, women ≥18 with confirmation of a negative pregnancy test; or 50 years without menstruation for at least 2 month.
- Written informed consent to participate

## Exclusion criteria

Contraindication for cardiac CT

- Renal insufficiency (GFR <60ml/min)
- Known allergy to iodinated contrast agent
- Known Hyperthyroidsm
- Non-sinus rhythm
- Known premature ventricular or supraventricular beats
- Hypotension (systolic blood pressure <100mmHg)

Contraindication for CMR

- Implanted cardiac pacemaker or defibrillator
- Other implanted medical devices
- Claustrophobia

# Methods

## Cardiac Computed Tomography

A cardiac multislice CT will be performed in inspiration. All patients intravenous metoprolol (2–20 mg) (BelocZOK, AstraZeneka, UK), if necessary to achieve a target HR of <63 bpm prior to the start of the scan. Body-surface-area adjusted contrast medium (30 to 95 ml Ultravist, Bayer Schering Germany or Visipaque 320, 320 mg/ml, GE Heathcare, UK) is injected into an antecubital vein via an 18-gauge catheter at a flow rate of 3.5 to 5 ml/s followed by 50 ml saline solution. Bolus tracking is performed with a region of interest placed into the ascending aorta. All cardiac CT examinations are performed with a LightSpeed VCT XT scanner (GE Healthcare) with prospective ECG triggering (SnapShot Pulse, GE Healthcare) or a Somaton Definition Flash scanner (Siemens). The images are transferred to an external workstation for postprocessing.

## 7.2 Echocardiography

Patients will be examined using a Philips iE33 echocardiography unit. A complete exam will be performed according to current guidelines of the division of echocardiography including contrast echocardiography. For the latter, Sonovue will be injected in 1 ml boli up to a total volume of 10 ml per patient. The contrast images will be obtained at a mechanical index of 0.4 or lower.

The images are transferred to an external workstation for analysis.

**7.3 Cardiovascular Magnetic Resonance**

All CMR exams will be performed at 1.5-T (Philips, Amsterdam, Netherlands). Contrast medium (2ml/10kg total) is injected into an antecubital vein via an 18-gauge catheter at a flow rate of 4ml/s followed by 30 ml saline solution. Then steady-state free precession cine images will be acquired in three long-axis views, planned on short-axis pilots at 60° angles to each other to visualize all segments of the heart. The images are transferred to an external workstation for postprocessing.

In all three imaging modalities the ratio of non-compacted to compacted myocardium will be analyzed in a 17 segment modell.

# ASSESSMENT OF SAVETY

Medications used in the context of cardiac CT are routinely administered by dedicated nuclear physicians or cardiologists. Our CT protocol mandates that in all patients who receive any type of imaging study–related medication, blood pressure measurements are performed before the administration of the first drug. Patients must not operate machinery (eg, drive) for 3 hours after the intravenous administration of 2-20 mg metoprolol (betablocker). Owing to our observance of this protocol, despite our high volume of cardiac CT examinations, we have not experienced adverse events, except in rare cases of transient hypotension.

Contrast material is generally well tolerated although approximately 1% of patients who receive low-osmolar nonionic contrast material can develop anaphylaxis symptoms, however most anaphylactic reactions are mild and non-allergic. The risk for serious or severe reactions—that is, anaphylaxis grade 3—has been estimated to be from 0.02% to 0.04% with our nonionic contrast material.

Adverse reactions to the echocardiography or CMR contrast are rare; up to 1% of patients develop a mild anaphylactic reaction, and, similar to CT, severe reactions of this type are les frequent than mild ones.

If any allergic reaction occur treatment and monitoring will be provided immediately (H1 antihistamines, corticosteroids, and H2-receptor blockers, epinephrine by our physician team. However, concerning this study, no harm or dangers in short term and long term are to be expected.

# 9 STATISTICS

All data will be presented as mean ± standard deviation. Nominal data will be tested using the chi-square test. Continuous data will be analyzed using analysis of variance to establish differences between IVNC and the remaining groups individually. A p value of <0.05 will be considered statistically significant. We will use receiver operating characteristics to generate cut-off values for optimized sensitivity and specificity to distinguish IVNC from all other groups of subjects.

According to the advice of our statistician (Prof. B. Seifert) we will include 20 patients with documented IVNC and 20 patients with no IVNC but a diagnosis which constitutes a differential diagnosis to IVNC, such as LV hypertrophy secondary to hypertension or aortic stenosis, concentric/dilated cardiomyopathy, competitive athletes with LV hypertrophy and patients without a history of cardiovascular disease who underwent cardiac CT due to the clinical suspicion of coronary artery disease. We hypothesize that we correctly identify all patients with IVNC due to the high spatial resolution of modern CT scanners, leading to an adequate 95%- confidence interval of 83%-100%.

# 10 Duties ON THE PART of the investigator

The investigator and sponsor confirms that this study is conducted in accordance with this protocol, “Good Clinical Practise” and the currently valid legal provisions. The PI is aware of his responsibilities and accepts them, accordingly.

Any severe adverse reactions, changes (to the protocol) will be reported promptly to the ethic committee.

For quality controlling Mr R. Milovanovic will monitor this study at the University Hospital of Zurich. He has extensive knowledge and experience in the field of good clinical practice (GCP). For a good quality assessment we plan cumulative 3 days for monitoring the correct documentation of all patients´ data, the CFR and the observance of the study plan according to the GCP guidelines.

# 11 ETical Considerations

Before enrollment, patients will have to obtain and sign written informed consent. The study subject can step down from study participation at any time without reasoning.

Participating patients will not primarily benefit form this study, nor any disadvantages are expected. However, the results of this study can contribute to a better understanding of the characterization of IVNC.

# 12 QualitY AND QUALITY ASSURANCE

All study related results will be used within the scope of the project. All data will be anonymised, the code list will kept locked up at the University Hospital Zurich, C HOF, room 117 and the images will be stored in the TriPacs and XCELERA system.

# Insurance

The insurance of the University Hospital Zurich will cover any study-related health damage (certification number 9.730.682; signed 19.11.2010).

# SIGNATURE PAGE

**Signature page for sponsor - investigator**

**Study Title: Cardiac Computed Tomographie: Characteristics of Isolated Ventricular Non-compaction**

**Approved by the following:**

|  |  |  |  |
| --- | --- | --- | --- |
|  | Date |  | Signature |

# REFERENCES
